# Supplementary material for: Innervation of supraclavicular adipose tissue: A human cadaveric study
Source: PLoS One. 2020 Jul 23;15(7):e0236286. doi: 10.1371/journal.pone.0236286 (PMC7377457; doi:10.1371/journal.pone.0236286)
Supplement: S1 File — (PDF) [file pone.0236286.s001.pdf]

Innervation of supraclavicular adipose tissue: A human cadaveric study
Supporting information.

**S1 Table. Adipocyte cross sectional area.**

| Adipocytes from axilla (Ax) cross sectional area | Adipocytes from supraclavicular region (Sc) cross sectional area |
|---|---|
| 4208 | 1805 |
| 4745 | 1883 |
| 4395 | 1406 |
| 2081 | 2021 |
| 3213 | 1344 |
| 7041 | 2378 |
| 3207 | 2430 |
| 3377 | 1525 |
| 3599 | 1512 |
| 2623 | 1776 |
| 3328 | 920 |
| 3140 | 1492 |
| 2549 | 2040 |
| 2283 | 1204 |
| 2338 | 1218 |
| 5369 | 1369 |
| 5474 | 892 |
| 1821 | 683 |
| 2421 | 655 |
| 2379 | 1455 |
| 3495 | 832 |
| 2166 | 523 |
| 2957 | 808 |
| 4355 | 1353 |
| 2975 | 2658 |
| 1683 | 388 |
| 3523 | 1875 |
| 1156 | 648 |
| 1146 | 572 |
| 1689 | 1346 |
| 3142 | 552 |
| 1662 | 1752 |
| 1081 | 813 |
| 2715 | 783 |
| 2677 | 516 |

| | |
|---|---|
| 1625 | 840 |
| 2094 | 1020 |
| 1822 | 606 |
| 2598 | 1575 |
| 1720 | 1223 |
| 2662 | 952 |
| 2882 | 1094 |
| 1286 | 1602 |
| 2271 | 850 |
| 2088 | 844 |
| 1630 | 1213 |
| 2241 | 609 |
| 1630 | 936 |
| 1324 | 1195 |
| 3304 | 1504 |
| 1707 | 2220 |
| 1649 | 881 |
| 6228 | 2316 |
| 3435 | 1427 |
| 2457 | 1250 |
| 1024 | 1735 |
| 2537 | 1523 |
| 3436 | 875 |
| 2551 | 2416 |
| 4370 | 1150 |
| 3058 | 1811 |
| 1562 | 989 |
| 2744 | 727 |
| 2608 | 1058 |
| 3098 | 1138 |
| 3015 | 1496 |
| 3304 | 1397 |
| 4070 | 1105 |
| 2523 | 1189 |
| 3578 | 1571 |
| 3301 | 1233 |
| 3187 | 829 |
| 1792 | 1341 |
| 4179 | 1256 |
| 2324 | 688 |

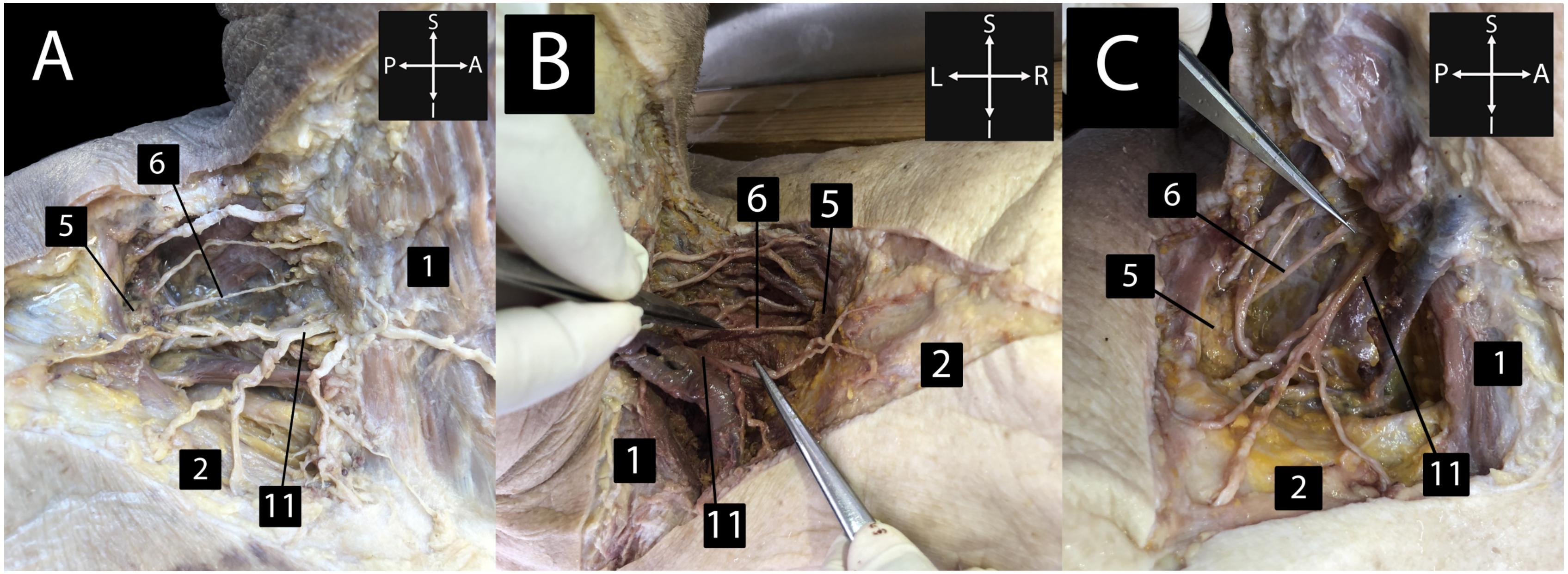

**S1 Fig. Representative images of dissected specimens.** (A) Cadaver 2 right hand side. (B) Cadaver 3 left hand side. (C) Cadaver 3 right hand side. 1 = sternocleidomastoid muscle, 2 = Clavicle, 5 = supraclavicular adipose tissue, 6 = nerve to supraclavicular adipose tissue, 11 = supraclavicular nerve.
